# Establishment of a Monoclonal Antibody against Human NTCP That Blocks Hepatitis B Virus Infection

**DOI:** 10.1128/jvi.01686-21

**Published:** 2022-03-09

**Authors:** Toshitada Takemori, Akiko Sugimoto-Ishige, Hironori Nishitsuji, Yushi Futamura, Michishige Harada, Tomomi Kimura-Someya, Takehisa Matsumoto, Teruki Honma, Miho Tanaka, Masami Yaguchi, Kyoichi Isono, Haruhiko Koseki, Hiroyuki Osada, Daiki Miki, Takashi Saito, Takashi Tanaka, Takehiro Fukami, Toshio Goto, Mikako Shirouzu, Kunitada Shimotohno, Kazuaki Chayama

**Affiliations:** a Drug Discovery Antibody Platform Unit, RIKEN Center for Integrative Medical Sciences, Yokohama, Japan; b Laboratory for Inflammatory Regulation, RIKEN Center for Integrative Medical Sciences, Yokohama, Japan; c Department of Clinical Laboratory, Tokyo Shinagawa Hospital, Tokyo, Japan; d Department of Virology and Parasitology, Fujita Health University School of Medicine, Toyoake, Japan; e Chemical Biology Research Group, RIKEN Center for Sustainable Resource Science, Wako, Japan; f Drug Discovery Structural Biology Platform Unit, RIKEN Center for Biosystems Dynamics Research, Yokohama, Japan; g Drug Discovery Computational Chemistry Platform Unit, RIKEN Center for Biosystems Dynamics Research, Yokohama, Japan; h Laboratory Animal Center, Wakayama Medical University, Wakayama, Japan; i Laboratory for Developmental Genetics, RIKEN Center for Integrative Medical Sciences, Yokohama, Japan; j Department of Gastroenterology and Metabolism, Graduate School of Biomedical and Health Sciences, Hiroshima University, Hiroshima City, Japan; k Research Center for Hepatology and Gastroenterology, Hiroshima University, Hiroshima City, Japan; l RIKEN Program for Drug Discovery and Medical Technology Platforms, RIKEN Cluster for Science, Technology and Innovation Hub, Yokohama, Japan; m Genome Medical Sciences Project, National Center for Global Health and Medicine, Ichikawa, Japan; n Collaborative Research Laboratory of Medical Innovation, Graduate School of Biomedical and Health Sciences, Hiroshima University, Hiroshima, Japan; o RIKEN Center for Integrative Medical Sciences, Yokohama, Japan; University of Southern California

**Keywords:** hepatitis B virus, monoclonal antibodies

## Abstract

Hepatitis B virus (HBV) infects 240 million people worldwide. Current therapy profoundly suppresses HBV replication but requires long-term maintenance therapy. Therefore, there is still a medical need for an efficient HBV cure. HBV enters host cells by binding via the preS1 domain of the viral L protein to the Na^+^/taurocholate cotransporting polypeptide (NTCP). Thus, NTCP should be a key target for the development of anti-HBV therapeutics. Indeed, myrcludex B, a synthetic form of the myristoylated preS1 peptide, effectively reduces HBV/hepatitis D virus (HDV) infection and has been approved as Hepcludex in Europe for the treatment of patients with chronic HDV infection. We established a monoclonal antibody (MAb), N6HB426-20, that recognizes the extracellular domain of human NTCP and blocks HBV entry *in vitro* into human liver cells but has much less of an inhibitory effect on bile acid uptake. *In vivo*, administration of the N6HB426-20 MAb prevented HBV viremia for an extended period of time after HBV inoculation in a mouse model system without strongly inhibiting bile acid absorption. Among the extracellular loops (ECLs) of NTCP, regions of amino acids (aa) 84 to 87 in ECL1 and aa 157 to 165 near ECL2 of transmembrane domain 5 are critically important for HBV/HDV infection. Epitope mapping and the three-dimensional (3D) model of the NTCP structure suggested that the N6HB426-20 MAb may recognize aa 276/277 at the tip of ECL4 and interfere with binding of HBV to the region from aa 84 to 87. In summary, we identified an *in vivo* neutralizing NTCP-targeting antibody capable of preventing HBV infection. Further improvements in efficacy of this drug will pave the way for its clinical applications.

**IMPORTANCE** A number of entry inhibitors are being developed to enhance the treatment of HBV patients with oral nucleoside/nucleotide analogues (NA). To amplify the effectiveness of NA therapy, several efforts have been made to develop therapeutic MAbs with neutralizing activity against HBs antigens. However, the neutralizing effect of these MAbs may be muted by a large excess of HBsAg-positive noninfectious particles in the blood of infected patients. The advantage of NTCP-targeted HBV entry inhibitors is that they remain effective regardless of viral genotype, viral mutations, and the presence of subviral particles. Although N6HB426-20 requires a higher dose than myrcludex to obtain equivalent suppression of HBV in a model mouse system, it maintained the inhibitory effect for a long time postadministration in proportion to the half-life of an IgG MAb. We believe that further improvements will make this antibody a promising treatment option for patients with chronic hepatitis B.

## INTRODUCTION

Hepatitis B virus (HBV) is a hepatotropic virus that infects 240 million people worldwide (1). HBV infection can lead to a wide range of liver pathologies, including fulminant hepatic failure, chronic hepatitis, cirrhosis, and hepatocellular carcinoma. Current therapies include antiviral agents that act directly on viral replication (2), such as nucleoside or nucleotide analogues that target HBV polymerase and interferon (IFN) therapy using pegylated IFN (peg-IFN) alpha (3), which mediates epigenetic repression of HBV covalently closed circular DNA (cccDNA) transcriptional activity (3, 4). Such treatments can suppress viral load but require long-term maintenance therapy (2). On the other hand, peg-IFN alpha therapy is contraindicated in certain subgroups of patients and is often associated with serious side effects (5). Thus, there remains an urgent medical need for an efficient HBV cure.

Most chronic HBV infections are acquired via maternal–infant transmission at birth or during infancy and childhood. Transmission to children can also occur from other close relatives who are HBV positive. On the other hand, most adults infected with HBV recover; however, approximately 10% of patients are unable to clear the virus.

From the immune prophylaxis viewpoint, effective and safe vaccines that can prevent HBV infection are available and are key protective measures. However, spontaneous mutation of the HBs antigen amino acid (aa) 145, from G to R, can lead to escape from the protective effect of the HBV vaccine (6), suggesting that vaccine-resistant HBV mutants can emerge during the period of persistent infection.

Although vaccine-acquired polyclonal antibodies have been shown to have a protective effect against HBV genotypes not included in the vaccine (7), the administration of higher doses of the vaccine-acquired antibodies to individuals infected with nonvaccine genotypes is required in some cases (8). Taken together, these findings show that there is a clear requirement for the development of new strategies to prevent and treat HBV and HDV infection.

The HBV envelope contains a membrane lipid bilayer into which small (S-HBsAg), middle (M-HBsAg) and large (L-HBsAg) envelope glycoproteins are integrated (9). L-HBsAg contains N-terminal preS1, central preS2, and C-terminal S domains. M-HBsAg is shorter than L-HBsAg and lacks preS1, and S-HBsAg consists only of the S domain. The S domain forms the antigenic loop (AGL) that participates in viral entry in addition to preS1.

For HBV/HDV coinfection, the HBV envelope proteins can also package the HDV ribonucleoprotein (RNP) (10), leading to the formation of HDV virions. Entry of HBV and HDV into a host cell requires virus attachment to cell surface heparan sulfate (HS) proteoglycans (HSPGs) through the AGL (11). This is followed by attachment of the myristoylated preS1 domain of the HBV envelope L protein (preS1 peptide) to Na^+^/taurocholate cotransporting polypeptide (NTCP) receptor (12). The binding of the preS1 peptide to NTCP is an important early entry step for HBV and HDV infection (12–14).

The normal physiological function of NTCP is as the main uptake transporter of conjugated bile acids from the blood into the liver, a process in which it plays a crucial role (14–16). It has been shown using a [^3^H]taurocholate uptake assay that HBV preS1 peptide binding to human NTCP affects its substrate transporting function (17).

In order to prevent viral entry, recent studies have reported novel types of monoclonal antibodies (MAbs) targeting either the AGL (18, 19) or the preS1 domain in L-HBsAg (20). These MAbs showed potential promise in HBV prevention and treatment of chronic HBV infection in a mouse model.

HBV surface proteins not only are a component of the viral particle envelope but also assemble into noninfectious subviral particles (SVP) that lack the viral genome (21). The concentration of SVP in serum can be several orders of magnitude greater than the concentration of infectious viral particles (21, 22). This large number of SVP may act as a decoy, probably making it difficult to neutralize viral infections with HBV-specific antibodies (23; see also Discussion).

As NTCP could theoretically be the main target for the development of anti-HBV agents, HBV entry inhibitors are expected to confer neutralization activity regardless of viral genotype, viral mutations, or the presence of excess subviral particles in the environment. The first HBV/HDV entry inhibitor, the synthetic linear peptide myrcludex B, mimics the preS1 domain of the HBV envelope L protein (17, 24, 25). Binding of myrcludex B to NTCP inhibits HBV infection at low concentrations but also blocks the uptake of bile salts. However, the drug-induced suppression of HBV infection was achieved with a 50% inhibitory concentration (IC_50_) that was around 1,000 times lower than that needed for inhibition of bile acid uptake (25), suggesting that the plasma bile acid (BA) inhibitory effect can be avoided during treatment by using an appropriate dose of myrcludex B. Myrcludex B showed a strong transient decrease in plasma HDV RNA levels and alanine aminotransferase (ALT) normalization in phase II studies of HBV- and HDV-coinfected patients (26, 27).

Myrcludex B has recently been approved as Hepcludex in Europe and is now available for HDV-infected patients under the recommendation of a 2-mg subcutaneous injection per day (28). Hepcludex can be given alone or in combination with a nucleoside/nucleotide analogue for the treatment of the underlying hepatitis B virus infection (29).

In the present study, we intended to establish a new therapeutic MAb against NTCP that may allow for longer-lasting inhibition of HBV infection than peptides *in vivo*, leading to a more simplified treatment regimen.

## RESULTS

### Establishment of mouse anti-NTCP MAbs by immunization of NTCP KO mice with NTCP that retained its native topological structure.

NTCP is a multiple-transmembrane glycoprotein that spans the cellular membrane up to 9 times with small extracellular loops (14). The major isoform of murine NTCP contains 362 amino acids and shares 73.8% sequence similarity with human NTCP (hNTCP), with the nonhomologous residues spread across the entire molecule. Therefore, to establish mouse MAbs with specificity for hNTCP, we established *Slc10a1* (the gene encoding NTCP) knockout (KO) mice as the host for immunization with hNTCP (see Materials and Methods). A previous report suggested that *Slc10a1*^−/−^ mice had elevated serum BA levels and failure to gain weight early in life but that the weight recovered later as the BAs decreased. Renormalization of BA levels was accompanied by increased BA sulfation, which may help to detoxify and eliminate the overloaded BAs (30). We noticed that most *Slc10a1*^−/−^ mice had reduced body weights compared with wild-type (WT) littermates starting from postweaning; however, the weight of the KO mice equaled the weight of WT mice as they grew older (>8 to 10 weeks of age).

To prepare immunizing antigens and reagents for antibody screening by flow cytometry, we transduced Daudi lymphoma and HepG2 cell lines with a cDNA encoding human NTCP in a retroviral vector (31) and established Daudi-NTCP and HepG2-NTCP cell lines that both expressed NTCP on the cell surface and bound preS1 at significant levels (data not shown).

In addition, NTCP proteins were purified and reconstituted into liposomes consisting of egg phosphatidylcholine with the adjuvant lipid A (see Materials and Methods), which maintained the antigen in an intact conformation as an immunogen.

We immunized NTCP KO mice intraperitoneally 4 to 6 times at 2-week intervals, alternating between NTCP liposomes and NTCP^+^ transfectants in adjuvants (see Materials and Methods). To prepare hybridoma cell lines secreting IgG^+^ MAb, IgG^+^ B cells were enriched by MACS from the pooled spleens of immunized mice and fused with the mouse myeloma cell line P3U1 according to electrofusion protocols (32). Hybridoma cell lines were cultured in 96-well culture plates. Each culture supernatant was collected between 5 and 10 days after initial cultivation and analyzed by flow cytometry for binding to NTCP^+^ Daudi cells but not to the parental Daudi cells. Of 20,000 samples analyzed, 792 culture supernatants had specificity for NTCP (data not shown).

### The MAb N6HB426-20 inhibits HBV infection of NTCP^+^ hepatoma cell lines without significant inhibition of bile acid uptake.

**FIG 1:**
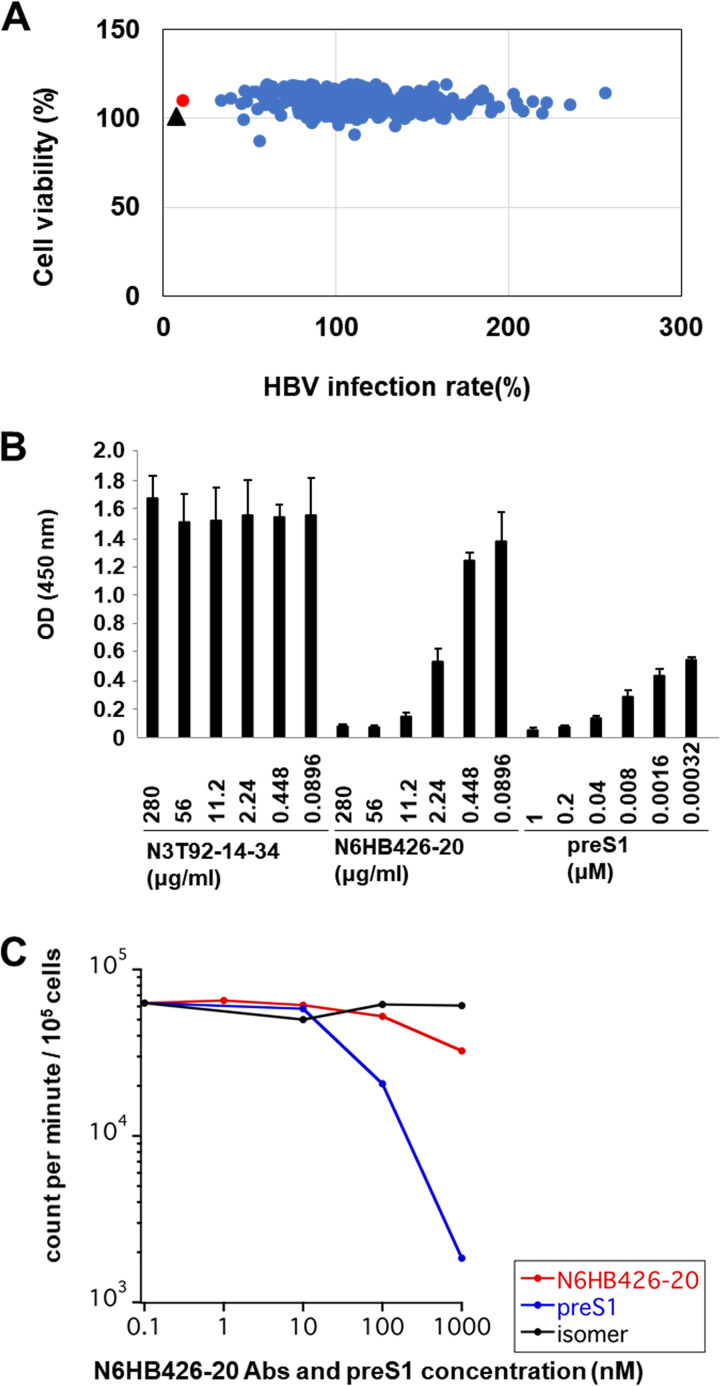
The NTCP MAb N6HB426-20 inhibits HBV entry *in vitro* with little inhibition of NTCP transport activity. (A) HepG2-NTCP-myc cells were pretreated with preS1 peptide or culture supernatant from hybridoma cell lines for 2 h and then infected with HBV/NL. At 8 days after infection, the HBV infection rate and the host viability were measured using a luciferase-based assay. Shown is a scatterplot of screening results. The *x* axis represents the HBV/NL infection rate. The *y* axis represents the viability of HepG2-NTCP-myc cells. A hit supernatant (N6HB426) is indicated by a red circle and preS1 activity by a black triangle. (B) HepG2 cell lines were stably transfected with human NTCP. The transfectants were incubated for 1 h with preS1 peptide, N6HB426-20 MAb, or N3T92-14-34 MAb as a control, followed by infection for 24 h with HBV in the presence of blocking reagents. At 24 h postinfection, cells were washed and cultured in fresh medium and the level of HBeAg in the culture supernatant was quantified by ELISA at 12 days after infection. (C) [^3^H]taurocholate uptake by hNTCP-HepG2 cells *in vitro* in the presence of N6HB426-20, preS1 peptide, or its inactive isomer, in which amino acids 11 and 13 were replaced by their respective d-enantiomers. NTCP^+^ HepG2 cell lines in a 48-well plate were preincubated in Na^+^ Ringer solution, followed by incubation with 1 μM [^3^H]taurocholate for 15 min at 37°C in the presence of N6HB426-20, preS1 peptide, or its isomer at different doses. Cells were washed and lysed in 1% Triton X-100, followed by mixing with liquid scintillation cocktail for liquid scintillation counting.

We analyzed the NTCP reactive culture supernatants for the ability to inhibit entry of a pseudo-hepatitis B virus expressing NanoLuc (HBV/NL) into HepG2 cells expressing NTCP-myc transfectants (HepG2-NTCP-myc cells). Detection of HBV/NL entry was monitored by NL activity (33). We observed that one culture supernatant, designated N6HB426, exhibited potent inhibitory activity, as did the soluble preS1 peptide (Fig. 1A). After limiting dilution of the N6HB426 hybridoma cell line twice, we established a clone, N6HB426-20, and then purified the IgG2a MAb from the culture supernatant.

As shown in Fig. 1B, to compare the neutralizing activity of N6HB426-20 MAb and myristoylated preS1 peptide, HepG2 cells were stably transduced with hNTCP. HBV (genotype D) was purified from the culture supernatant of HepAD38 cells (34) and used for infection under conditions (500 genomes/cell) that allowed approximately 20% of the cells to be infected (data not shown). The transfectants were infected with HBV in the presence of the N6HB426-20 MAb or a control MAb N3T92-14-34, and the level of HBeAg in the culture supernatant was quantified 12 days later. N3T92-14-34, which was shown to recognize methionine at aa 3 in the N-terminal region, binds NTCP but does not have neutralization activity (data not shown). The results show that the N6HB426-20 MAb suppressed HBV infection, with an IC_50_ of 1.52 μg/mL (equivalent to 10 nM based on the molecular mass of IgG). On the other hand, the myristoylated preS1 peptide showed a more profound inhibition, with an IC_50_ of <320 pM.

NTCP is the HBV entry receptor and also an uptake transporter of conjugated bile salts from the blood into the liver. A previous study showed that [^3^H]taurocholate uptake through NTCP could be specifically blocked by the myristoylated preS1 peptide of the L-HBsAg (13). As shown in Fig. 1C, we observed that the preS1 peptide suppressed [^3^H]taurocholate uptake by 70% already at 100 nM and >95% at 1,000 nM. In contrast, the N6HB426-20 MAb had almost no effect on the entry of [^3^H]taurocholic acid at 100 nM but reduced it by 40% at 1,000 nM, which is 100 times higher than the IC_50_ needed to reduce HBV entry. Thus, the N6HB426-20 MAb blocks HBV entry into target cells at a dose that does not significantly affect the absorption of bile acid.

### The MAb N6HB426-20 neutralizes HBV infection of primary human hepatocytes *in vitro*.

**FIG 2:**
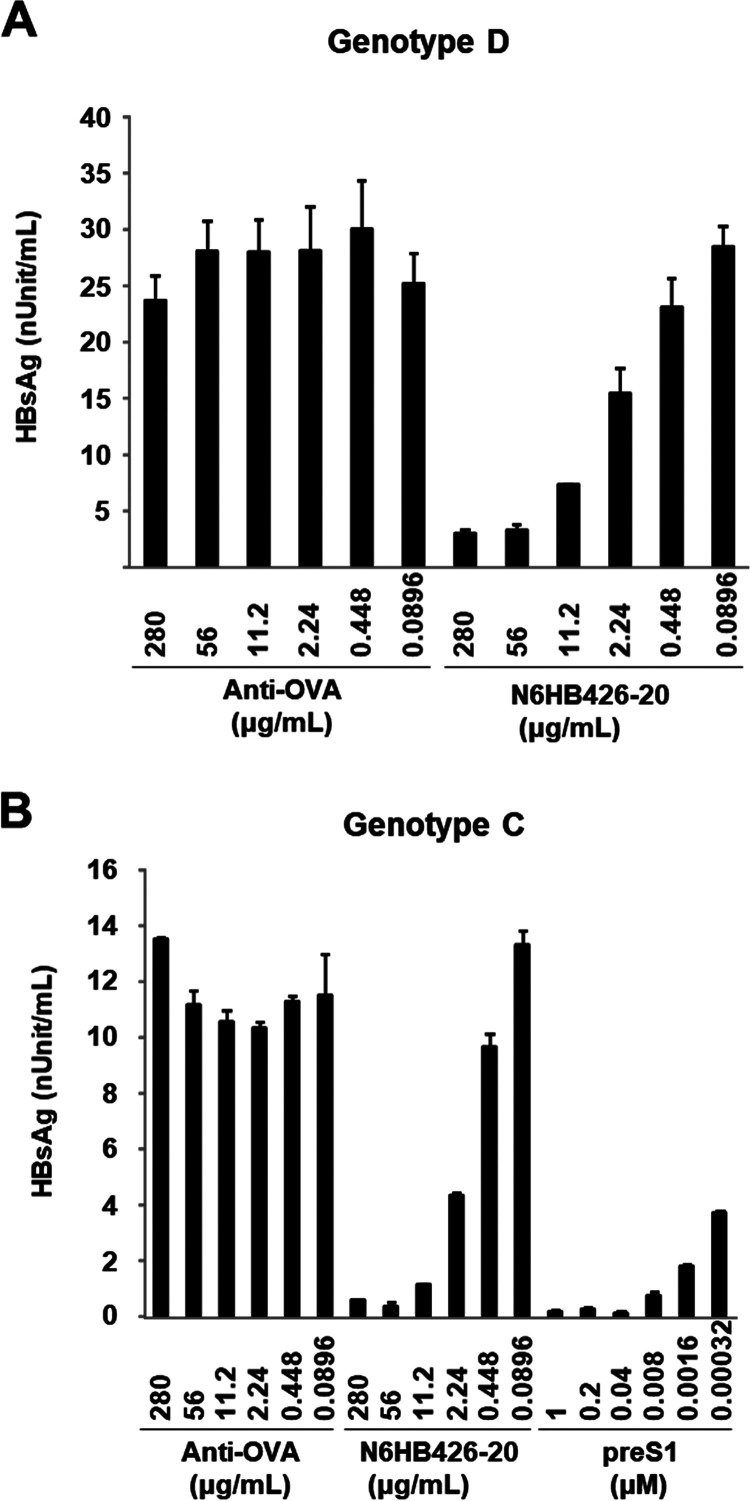
The N6HB426-20 MAb inhibits HBV infection *in vitro*. PXB cells were treated with anti-OVA MAb, N6HB426-20 MAb, or preS1 peptide for 1 h. After washing, cells were infected with HBV genotype D (A) or genotype C (B) derived from serum samples from HBV-infected patients, along with preS1 peptide or antibody. At 24 h postinfection, cells were washed and cultured in fresh medium. At 7 days postinfection, the level of HBsAg was measured by ELISA. The IC_50_ was calculated by KaleidaGraph 4.5 software (Synergy Software, PA).

Importantly, when primary human hepatocytes (PXB cells) were used as target cells for HBV infection, a finding similar to that in Fig. 1B was obtained (Fig. 2). N6HB426-20 efficiently blocked infection of PXB cells by HBV genotype D (Fig. 2A) as well as genotype C (Fig. 2B) derived from HBV-infected patients. Of note, N6HB426-20 effectively inhibited HBV infection, with an IC_50_ of ∼8 nM for HBsAg, whereas myristoylated preS1 peptide showed a more profound inhibition, with an IC_50_ of <320 pM (Fig. 2B).

### N6HB426-20 may recognize the fourth extracellular loop in NTCP.

Human NTCP is a multipass transmembrane protein and is thought to be comprised of an extracellular N terminus, 4 extracellular loops (ECL), and a cytoplasmic C-terminal domain (14). hNTCP has different residues from mouse NTCP at aa 84 to 87 in ECL1 and aa 157 to 165 in the transmembrane 5 domain downstream of ECL2, which are crucial for HBV infection (14).

To investigate the NTCP region to which N6HB426-20 binds, a parental plasmid expressing NTCP was used as a template to make alanine mutations in the extracellular loops. Each of 28 distinct mutants was individually transfected into human Huh7 cells, and transfectants were infected *in vitro* with recombinant HBV encoding NanoLuc in the absence or presence of N6HB426-20 (data not shown). We then searched for mutants that could be infected with HBV/NL but were not blocked by the N6HB426-20 MAb.

**FIG 3:**
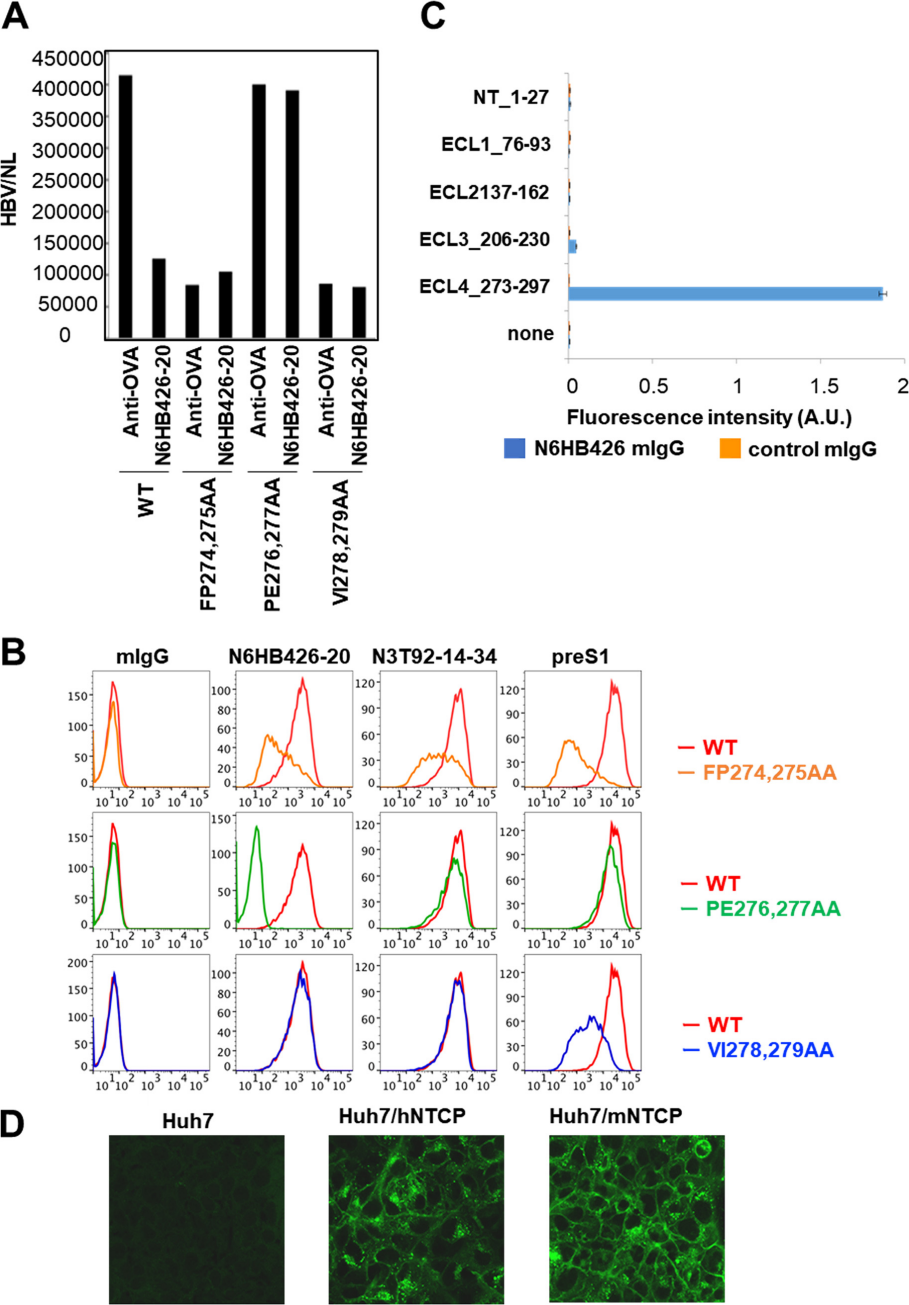
N6HB426-20 epitope mapping. (A) Huh7 cell lines transfected with WT or NTCP mutants (F274A/P275A, P276A/E277A, or V278A/I279A) were infected with HBV/NL *in vitro* in the presence or absence of the N6HB426-20 MAb or anti-OVA MAb, as a control. At 6 days after infection, the level of NanoLuc activity was measured using a Nano-Glo luciferase assay system. Experiments were carried out twice. (B) Huh7 cells were transiently transfected with myc-tagged WT NTCP or NTCP with alanine mutants at aa 274/275 (top row), 276/277 (middle row), and 278/279 (bottom row). Transfectants were stained with N6HB426-20 (10 μg/mL), N3T92-14-34 (1 μg/mL), normal mouse IgG (10 μg/mL), or preS1^Alexa Fluor 647^ (0.1 μg/mL). The staining with mouse IgG antibodies was developed with allophycocyanin (APC)-conjugated anti-mouse IgG (BioLegend). Experiments were carried out twice. (C) Synthetic peptides were designed according to the sequences of predicted regions of extracellular loops (N terminus, aa 1 to 27; ECL1, aa 76 to 93; ECL2, aa 137 to 162; ECL3, aa 206 to 230; ECL4, aa 273 to 297; see Materials and Methods). Binding ability of the N6HB426-20 antibody to the synthetic peptides was measured by ELISA on a peptide-captured 96-well plate. Each value represents the mean and SD of 4 wells. Experiments were carried out twice. (D) N6HB426-20 recognizes human and mouse NTCP. Huh7 cell lines expressing human or mouse NTCP were fixed and stained with N6HB426-20, developed with anti-mouse IgG^FITC^ and analyzed by confocal microscopy.

As shown in Fig. 3A, the transfectant expressing the P276A/E277A mutation in ECL4 of NTCP was infected with HBV/NL as efficiently as the parent strain, but the infection was not inhibited in the presence of the N6HB426-20 MAb. PreS1^Alexa647^ and a control N3T92-14-34 MAb stained the P276A/E277A mutant cell line to an extent similar to that of the parental cell line (Fig. 3B), indicating that this mutation did not affect NTCP cell surface expression. On the other hand, the N6HB426-20 MAb efficiently stained the parental cell line but lost binding activity to the P276A/E277A mutant cell line, suggesting that the N6HB426-20 MAb recognized aa 276/277 in ECL4. This possibility was supported by the results in Fig. 3C showing that N6HB426-20 bound to a synthetic peptide corresponding to ECL4 (aa 273 to 297) but not to peptides corresponding to other extracellular loops.

The F274A/P275A mutation reduced the binding affinity of the N6HB426-20 MAb, N3T92-14-34 MAb, and preS1 and suppressed HBV infection, suggesting that this mutation affected the topological structure of NTCP. The F278A/P279A mutation interfered with HBV infection and preS1 binding but not with N6HB426-20 binding.

Amino acid residues 84 to 87 in ECL1 of hNTCP are the critical determinants restricting entry of HBV and HDV (14, 35, 36). It has been reported that the substitution of aa 84 to 87 (Arg-Leu-Lys-Asn) of hNTCP with the mouse counterpart (His-Leu-Thr-Ser) significantly decreased viral infection (14, 36). We observed by fluorescence-activated cell sorting (FACS) analysis that this substitution in hNTCP did not affect the binding activity of the N6HB426-20 MAb (data not shown). Together, these results suggest that the amino acid residues at positions 276/277 in the ECL4 of NTCP are responsible for N6HB426-20 binding. Since the epitope recognized by N6HB426-20 has an amino acid sequence common to mice and humans (14), this MAb recognizes both human and mouse NTCP (Fig. 3D).

### A three-dimensional model of NTCP.

We performed a computational approach using homology modeling to understand the topological basis of the NTCP structure. For construction of the NTCP model, we searched for homologous templates and selected the crystal structure of a sodium bile acid symporter from Yersinia frederiksenii (PDB code 4N7W) because it shared the highest identity (22.1%) with the hNTCP amino acid sequence and insertion/deletion status. Based on Molecular Operating Environment (MOE) 2019.0102 (Chemical Computing Group, Montreal, Quebec, Canada), homology modeling was performed and the highest-scoring model was adopted.

**FIG 4:**
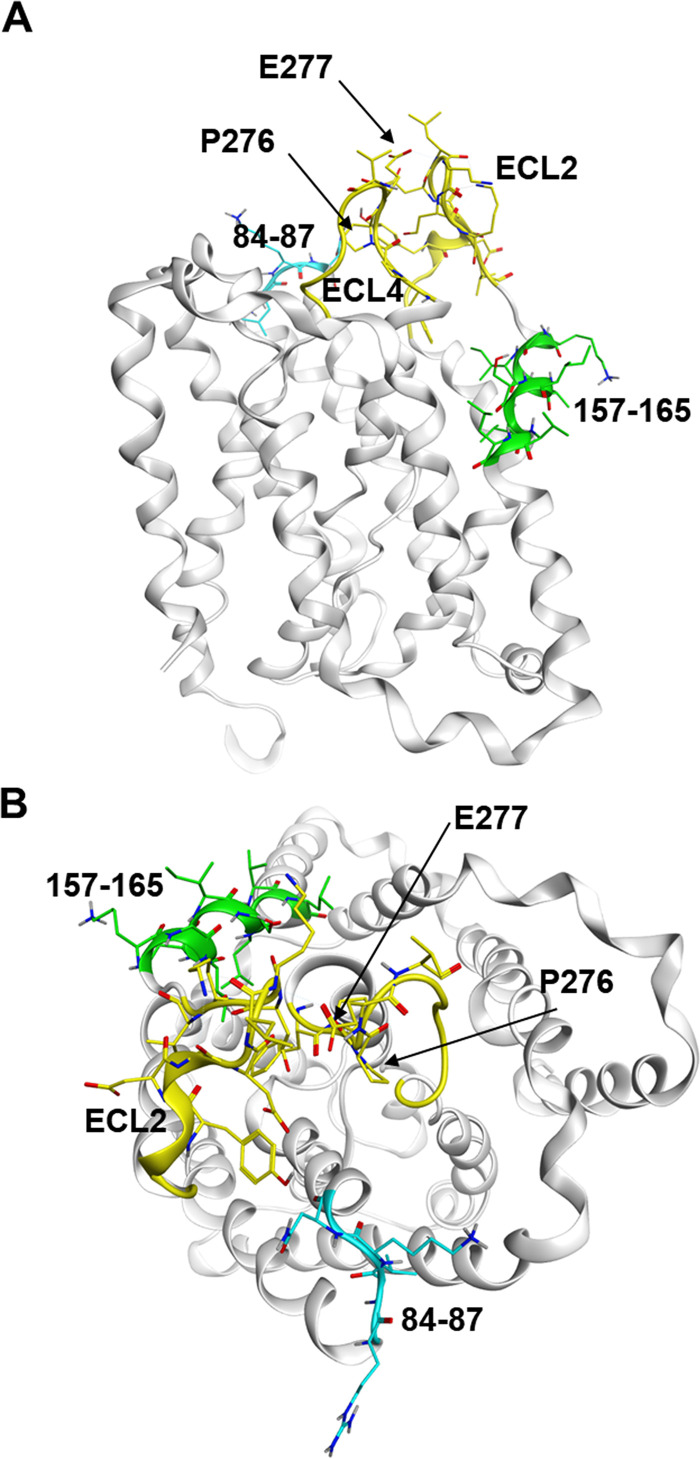
3D structural model of NTCP: side view (A) and top view (B). The NTCP homology model was constructed based on the crystal structure of a sodium bile acid transporter (PDB code 4N7W). The extracellular loops ECL2 and ECL4 in NTCP, which include the binding sites for N6HB426-20 MAb, are colored yellow. The important regions for HBV entry (aa 84 to 87 and aa 157 to 165) are highlighted in cyan and green, respectively.

As shown in Fig. 4A, the model suggests that ECL2 and ECL4 form loop structures on the surface of NTCP. P276/E277 are located at the tip of ECL4 (yellow) and protrude onto the surface of the molecule, confirming that P276/E277 may be in a position recognizable by the N6HB426-20 MAb. The HBV binding site in ECL1 (aa 84 to 87, cyan) appears to be exposed on the extracellular surface, as previously predicted (14, 35). On the other hand, the preS1 binding site (aa 157 to 165, green) is localized to transmembrane domain 5 downstream of ECL2.

As shown in Fig. 4B, when the NTCP model is viewed from the extracellular side, P276/E277 recognized by the N6HB426-20 MAb is located between the key motifs of HBV infection at aa 84 to 87 (cyan) and aa 157 to 165 (green). Therefore, we predict that N6HB426-20 binding to P276/E277 may sterically interfere with HBV binding to NTCP.

### Administration of N6HB426-20 to human liver chimeric mice inhibits HBV infection.

**FIG 5:**
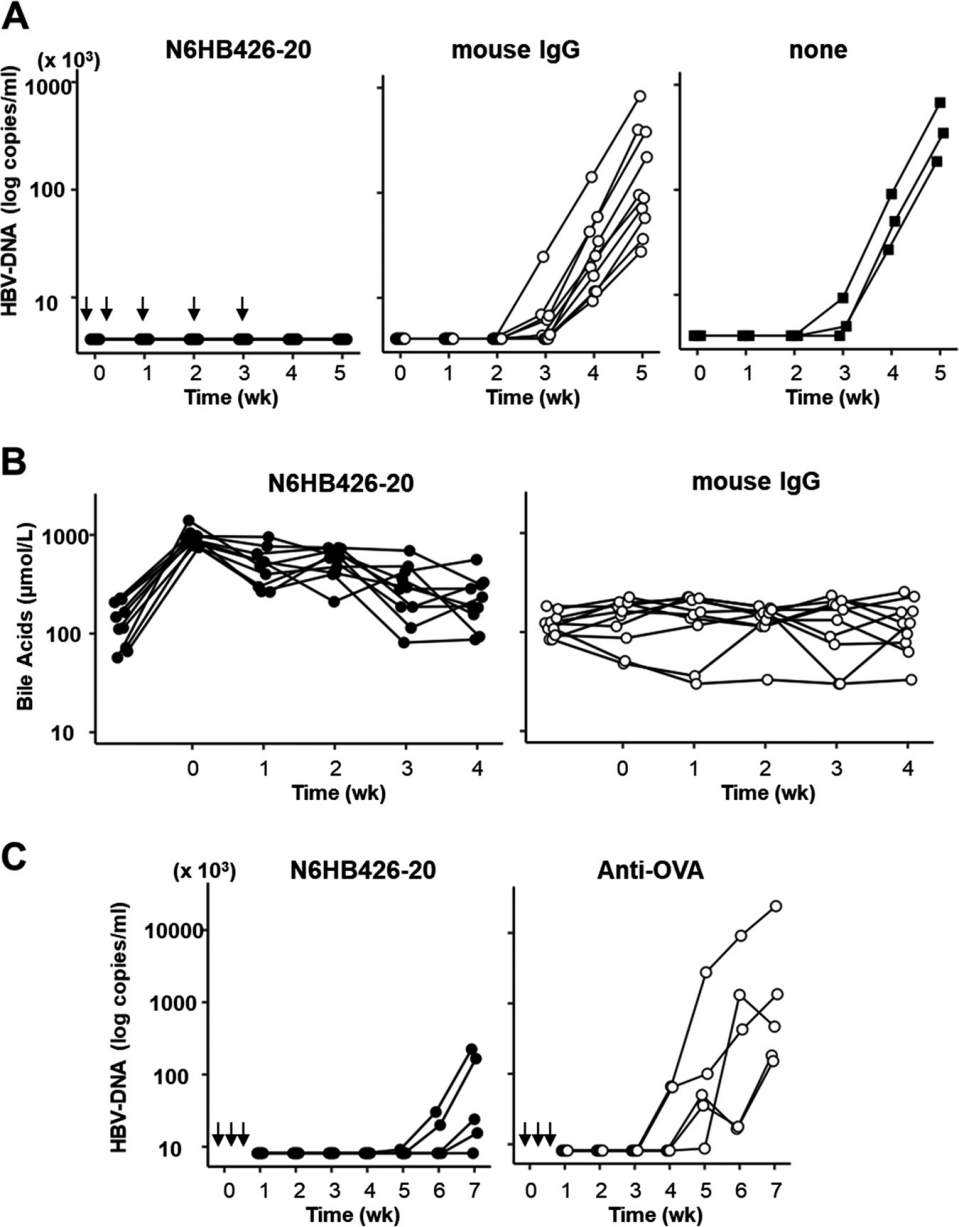
The N6HB426-20 MAb inhibits virus infection *in vivo*, accompanied by a temporary elevation in serum bile acids. (A) Time course studies in 20 uPA SCID mice inoculated with human serum samples positive for HBV, with administration of the N6HB426-20 MAb (IgG2a/κ, *n* = 10 [left]) or normal mouse IgG (*n* = 10 [middle]). Fifty microliters of human serum containing 10^3^ copies of HBV was injected intravenously into each mouse. Two milligrams of N6HB426-20 MAb or normal mouse IgG was administered 1 day before (day −1) and 2 days after (day 2) virus inoculation, followed by intraperitoneal injection of 1 mg of each antibody at 1, 2, and 3 weeks after virus inoculation. A time course in three mice without treatment to confirm infectivity of the HBV positive serum is shown as “none” in the right graph. The HBV DNA serum level was measured at each indicated time point. HBV DNA was quantified by real-time PCR using DNA extracted from 10 μL of mouse serum. The lower quantitation limit of this assay is 3.61 log copies/mL. (B) Serum bile acid concentration was measured at each time point from 10 μL of mouse serum using an enzyme cycling assay. (C) Time course studies in 10 uPA SCID mice inoculated with human serum samples positive for HBV, with administration of 1 mg of N6HB426-20 MAb (left) or anti-OVA mouse MAb (IgG1/κ) (right) in each recipient (40 mg/kg). Fifty microliters of human serum containing 10^3^ copies of HBV was injected intravenously into each mouse. The N6HB426-20 MAb or anti-OVA MAb were administered 1 day before and after and 4 days after virus inoculation. The HBV DNA serum level was measured as in panel A.

As shown in Fig. 5A, we analyzed the activity of N6HB426-20 in preventing virus infection *in vivo* using urokinase-type plasminogen activator/severe combined immunodeficiency (uPA^+/+^ SCID^+/+^) mice transplanted with human hepatocytes (37). The human liver chimeric mice were injected intravenously with 2.0 mg of either N6HB426-20 (*n* = 10) or normal mouse IgG, as a control (*n* = 10), 1 day before and 2 days after HBV inoculation, followed by intraperitoneal injection of each antibody (1 mg) at 1, 2, and 3 weeks after viral inoculation.

After inoculation, serum HBV DNA titers were determined weekly by quantitative real-time PCR. All 10 mice injected with the control IgG developed quantitatively measurable viremia by 4 weeks after HBV inoculation, and HBV DNA titers further increased at 5 weeks. In contrast, none of 10 mice injected with the N6HB426-20 MAb showed viremia at any time point after HBV inoculation. As shown in Fig. 5B, the level of serum bile acids was elevated 1 day after the first administration of the N6HB426-20 MAb but gradually decreased during additional administration of the MAb. Except for one mouse, the level was almost comparable to the level prior to MAb administration 1 week after the finial injection of MAb. Considering that the circulating half-life of IgG is roughly 10 to 21 days (38), these results suggest that viral production was inhibited by the N6HB426-20 MAb in chimeric mice at a dose lower than that required for inhibition of bile acid uptake by NTCP.

As shown in Fig. 5C, we further analyzed HBV inhibition by the N6HB426-20 MAb in chimeric mice while reducing the dose and frequency of injections. Three doses of N6HB426-20 (1 mg/mouse, 40 mg/kg of body weight) at 1 day before and 1 and 4 days after HBV inoculation (10^3^ copies) eliminated HBV viremia up to at least 5 weeks postinfection. In contrast, 5 mice injected with 1 mg of control IgG1 MAb developed quantitatively measurable viremia already at 4 to 5 weeks after infection. Therefore, even with a reduced dose and frequency of injections, administration of N6HB426-20 achieved suppression of the onset of viremia for 4 to 5 weeks after viral inoculation.

## DISCUSSION

In the present study, we established the MAb N6HB426-20, which recognizes the cell surface NTCP protein. Receptor recognition by the MAb is indistinguishable between mice and humans.

The MAb blocked *in vitro* HBV infection of hepatoma cell lines transfected with hNTCP at a dose that did not significantly affect the entry of [^3^H]taurocholic acid.

N6HB426-20 blocked HBV infection of HepG2/NTCP cells with an IC_50_ of 10 nM for HBeAg and blocked infection of genotype D and C HBV isolates in human hepatocytes with an IC_50_ of ∼8 nM for HBsAg. These results suggest that the inhibitory activity is less potent than the effect on HBV inhibition by myrcludex B, which blocks HBV infection with an IC_50_ of 669 pM for HBsAg and 83 pM for HBeAg in primary human hepatocytes (25).

NTCP contains 4 extracellular loops (ECL), and amino acids (aa) 84 to 87 in ECL1 and aa 157 to 165 in transmembrane 5 downstream of ECL2 are critically important for viral entry and substantially contribute to HBV and HDV infection (13, 14). Amino acids 84 to 87 are thought to be exposed on the extracellular surface and to be directly involved in HBV binding (14, 35). Based on the NTCP model, the present study suggests that the N6HB426-20 MAb can access and recognize amino acids P276/E277 in ECL4, which are located at the tip of ECL4 and protrude into the extracellular space (Fig. 4). Therefore, the binding of the N6HB426-20 MAb to NTCP is likely to inhibit the binding of HBV to aa 84 to 87.

To evaluate the efficacy of N6HB426-20 against HBV infection *in vivo*, we used human hepatocyte chimeric mice that developed viremia within 4 weeks after HBV inoculation and which was further exacerbated a week later. HBV production was inhibited for at least 5 weeks after virus inoculation by administration of 80 mg/kg of body weight (2 mg per mouse) of N6HB426-20 before and 2 days after viral inoculation, followed by 40 mg/kg of antibodies at weekly intervals. The level of plasma bile acids was elevated 1 day after N6HB426-20 administration, but the level had returned to nearly normal 3 weeks later, in line with restoration of NTCP-mediated bile acid transport. At this time point, viral production was continuously decreasing, suggesting that N6HB426-20 can achieve inhibition of HBV infection at a dose lower than the concentration required for inactivation of NTCP cellular function.

Bile acids are synthesized in the liver and stored in the gallbladder. After food intake, they are released into the small intestine to promote the digestion and absorption of nutrients. They are reabsorbed into the enterocytes in the terminal ileum by the apical sodium-dependent bile acid transporter (ASBT; *SLC10A2*) and released into the portal circulation, where they are returned directly back to the liver via the portal vein. Bile acid is absorbed on the basolateral side of the hepatocyte, mainly by NTCP and to a lesser extent by members of the organic anion transporting polypeptide (OATP) family, OATP1B1 (*SLCO1B1*) and OATP1B3 (*SLCO1B3*). Both NTCP and OATP proteins are expressed on the sinusoidal membrane of hepatocytes, but they belong to different protein families (39, 40).

We believe that the MAb is unlikely to affect bile acid absorption by ASBT because N6HB426-20 is not expected to come into contact with the extracellular domain of ASBT, which faces the lumen of the small intestine. Since NTCP has little amino acid sequence homology with OATP1B1 and OATP1B3, it is also highly unlikely that N6HB426-20 will react with OATP1B1 or OATP1B3 to inhibit their absorption of bile acids. In addition, amino acids in the putative epitope region recognized by N6HB426-20 are not conserved in the predicted extracellular loops in OATP1B1 and OATP1B3 (data not shown).

Regarding the efficacy of myrcludex B in the mouse model, it has been previously reported that administration of the peptide (2 mg/kg) 1 h before HBV and HVD infection followed by once per day in the first 4 days after infection resulted in efficient suppression of HBV and HDV infection, analyzed at 4 weeks after infection (41). When conducting a study under similar conditions, we observed that 3 doses of N6HB426-20 (40 mg/kg) 1 day before and after and 4 days after HBV infection abolished viral production at 5 weeks postinfection. The results indicate that N6HB426-20 requires a higher dose than myrcludex to obtain equivalent suppression of HBV in chimeric mice under similar conditions.

Key immune components to control HBV infection are antigen-specific T cell responses and neutralizing anti-HBs antibodies mediating HBsAg clearance and lifelong protective immunity (42, 43). The development of antibodies targeting HBsAg has recently been reported, such as mouse E6F6 and human Bc1.187 targeting the AGL (18, 19) and human 2H5-A14 with specificity for the preS1 domain in HBV large surface protein (LHB) (20).

A single injection of E6F6 and 2H5-A14 prior to virus inoculation had potent prophylaxis activity in model mice at a dose of 15 to 20 mg/kg. However, frequent antibody administration was required to obtain a significant effect on virus production in chimeric mice, such as twice-weekly administration of 2H5-A14 (20 mg/kg) and once-weekly administration of Bc1.187 (50 mg/kg) for 3 weeks. In the case of mouse MAb E6F6, a single injection of 20 mg/kg of antibody caused a significant decrease in HBV production that was maintained for an average of 14 days. In assessing these results, we need to note that the half-life of human-derived antibodies is shortened in a mouse environment.

In the blood of chronically infected patients, there are high levels of circulating SVP of HBV in the shape of spheres and filaments, at a level 100- to 1,000-fold higher than for complete virions (21). HBV antigen S is the predominant protein present in both virions and SVP, and L is enriched in virions and filaments but barely detectable in spheres (21, 44). It has been reported that SVP reduced the neutralizing effect of anti-HBs antibodies in HBV infection *in vitro*, whereas SVP had little effect on viral entry (23). Therefore, it remains to be verified whether the virus-neutralizing activity of these MAbs observed in model mice will be reproduced equally in infected patients in the presence of a large excess of subviral particles. In protected individuals, neutralizing Abs and/or memory B cells with a diversified V gene repertoire and biological properties appear to sustain anti-HBV immunity rather than a single high-quality antibody (19). Therefore, another concern is whether long-term monotherapy with a single neutralizing MAb poses the risk of developing escape mutants in patients with chronic hepatitis, resulting in treatment failure.

Current guidelines recommend continuing long‐term oral nucleoside/nucleotide analogue (NA) therapy until immunological control of HBV is established (45). Unfortunately, patients with chronic hepatitis B (CHB) typically exhibit ineffective immune responses against HBV. This state is thought to be due to a high titer of long-lasting serum HBsAg, which triggers HBV immunotolerance in CHB patients (46). Therefore, the ideal clinical outcome may involve recovery from immune tolerance associated with the loss of HBsAg, thus promoting immune recovery in the host. Antiviral treatment with NAs has no significant impact on the serum HBsAg levels because these particles are almost entirely derived from noninfectious HBV SVP (21, 22). Therefore, avoiding immune tolerance in chronically infected patients and inducing an effective anti-HBV immune response constitute a major challenge at present.

The advantage of targeting NTCP to block HBV invasion is that its effect can be exerted regardless of virus genotype, virus mutations, or the presence of a large excess of subviral particles in patients’ blood. Potential clinical applications of N6HB426-20 include postexposure prophylaxis or preventing vertical transmission or reinfection after liver transplantation in HBV-infected individuals. The N6HB426-20 MAb synergizes with human anti-HBs immunoglobulin (HBIg) and shows potent inhibitory activity for HBV infection *in vitro* with a smaller dose of MAb (H. Nishitsuji and K. Shimotohno, unpublished data). Therefore, when a neutralizing MAb is administered in combination with N6HB426-20, it is conceivable that a synergistic effect achieves the cooperative suppression of HBsAg and the stable suppression of virus production, regardless of the inhibitory effect of SVP.

Since residues 84 to 87 of hNTCP are involved in HBV and HDV infection (14, 36), He et al. established a MAb, 18D1, that recognizes aa 84 to 87 on hNTCP by immunization of mice with hNTCP transfectants and examined whether targeting the epitope could reduce HDV infection. The results showed that the 18D1 MAb suppressed HDV infection *in vitro* with an IC_50_ of ∼15 μg/mL (equivalent to 100 nM). In a mouse model, intraperitoneal injection of 18D1 (60 mg/kg) prior to viral inoculation partially inhibited the HDV infection at low efficiency. However, the effects on HBV infection and bile acid uptake are unknown.

The 18D1 MAb lost binding to hNTCP by replacement of the amino acid at position 86 by its murine counterpart, although this replacement did not affect HDV or HBV infection (14). This raises the possibility that 18D1 and HDV may react with different conformational epitopes within the region from aa 84 to 87.

NA mainly target the viral reverse transcriptase/polymerase and prevent the formation of new HBV DNA from pregenomic RNA (pgRNA). Although NA efficiently inhibit viral replication, residual viruses can be produced from chronically infected cells, so naive hepatocytes can be constantly infected. *De novo* HBV infection results in cccDNA formation from incoming relaxed circular DNA, but this step does not appear to require the activity of viral reverse transcriptase/polymerase. Therefore, NA have only minimal effects on the existing cccDNA pool and on newly formed cccDNA molecules from *de novo* infection (2, 47).

Although *de novo* HBV infection via NTCP may maintain the DNA pool, HBV entry inhibitors could help neutralize residual virus, protect naive hepatocytes from infection, and promote elimination of infected cells. Therefore, the combination of NA and antivirals may have a potent synergistic effect that may ultimately promote the elimination of infected cells. The benefit of combination therapy with HBV entry blocking and NA treatment could be seen in clinical trials using bulevirtide (Hepcludex), formerly myrcludex B (28). Bulevirtide at a 2-mg daily dose exerted an effective reduction of HDV RNA levels and improvement of liver inflammation in a combination with tenofovir disoproxil fumarate (TDF) that was much more potent than TDF monotherapy. A dose of 2 mg per day of bulevirtide does not completely block the NTCP-mediated absorption of bile acids (29).

Myrcludex B had a half-life of the peptide-receptor complex at the surface of primary human hepatocytes of about 11 h and accumulated in the liver after injection with a half-life of about 16 h in mice (48). It would be interesting to know the effect of a combination therapy with NA using the N6HB426-20 MAb, which has a longer half-life *in vivo*.

In summary, we propose that N6HB426-20, a mouse MAb, is a promising treatment option for patients with chronic hepatitis B infection. Concerning the therapeutic potential, we need to perform further studies to improve the efficacy of the neutralization and humanize the MAb. We believe that this development will pave the way for the establishment of stronger and better combination therapies for chronically infected patients.

## MATERIALS AND METHODS

### Generation of genome-edited mice.

**FIG 6:**
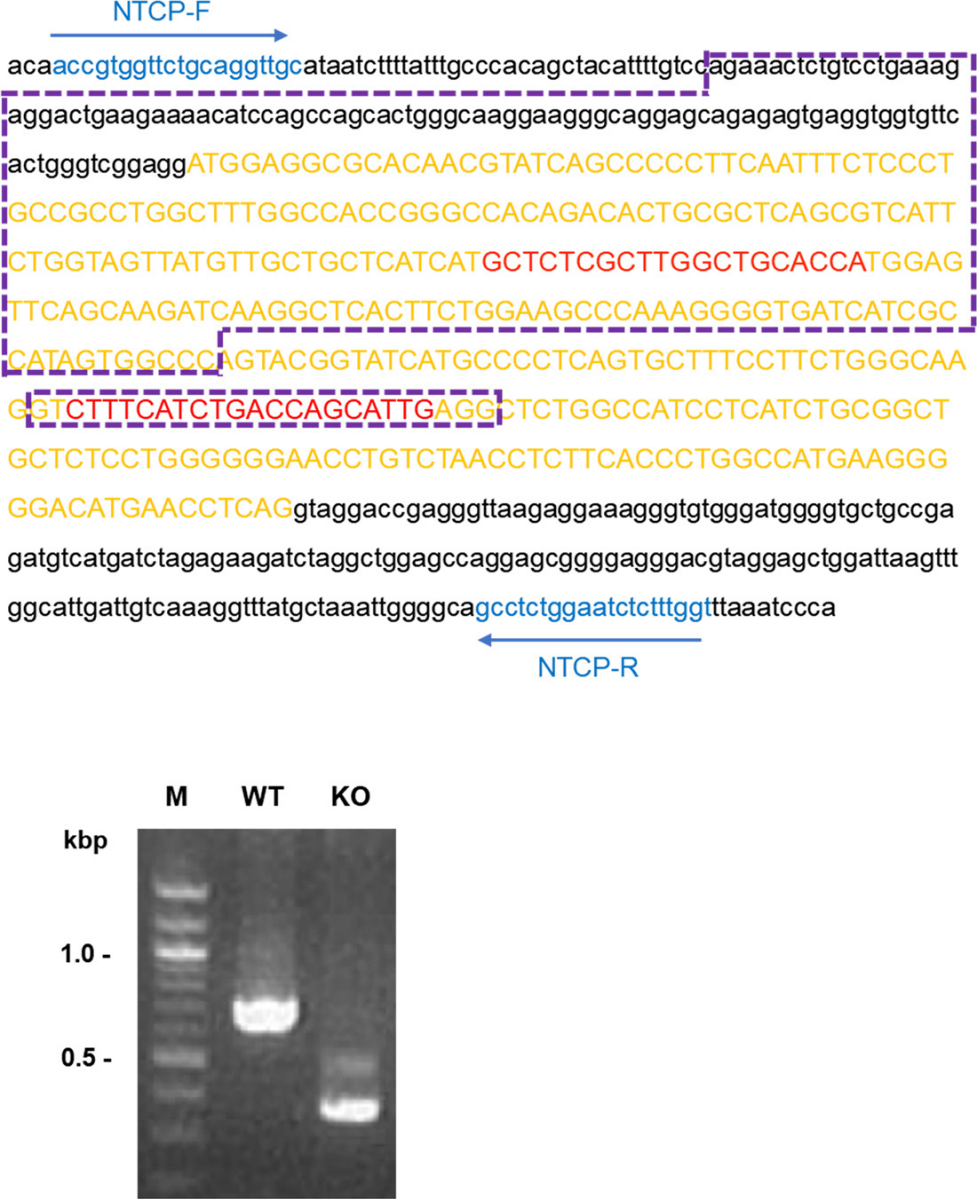
NTCP genome editing. (Top) The sequence of the first exon (capital orange letters) and introns (lowercase) of the *SLC10A1* (NTCP) gene. Genome editing was performed with two gRNA (red) in the exon at once; NTCP KO mice used in this study lack two separate sequences (302 bp and 25 bp) (dashed boxes). (Bottom) Genotyping PCR to confirm the *SLC10A1* KO was carried out using the primer set NTCP-F and NTCP-R (indicated by blue arrows in panel A) and tail DNA.

Genome editing of fertilized mouse embryos was performed according to a previously reported method (49). Briefly, a PCR-amplified fragment containing a T7 promoter and guide RNA (no. 1, GCTCTCGCTTGGCTGCACCA plus tracrRNA sequence; no. 2, CTTTCATCTGACCAGCATTG plus tracrRNA sequence) was cloned into pUC19. The Cas9 gene PCR product derived from pX330 was cloned downstream of the T7 promoter into another pUC19 plasmid. Single-stranded guide RNA and Cas9 mRNA were synthesized by *in vitro* transcription using these constructs and a MEGAshortscript T7 transcription kit (Thermo Fisher Scientific, Waltham, MA) and then purified using a MEGAClear kit (Thermo Fisher Scientific). Two guide RNAs (10 ng/μL each) and Cas9 mRNA (10 ng/μL) were microinjected into 100 C57BL/6N pronuclear stage embryos, and the next day, two-cell stage embryos were transplanted into pseudopregnant mice. Founder genome editing was determined by PCR/sequence analysis (Fig. 6).

### Plasmid vector construction.

A cDNA encoding human *SLC10A1* (NTCP) was purchased from Kazusa DNA Research Institute (IMAGE clone identifier, 100072498; Kazusa, Japan). The cDNA was amplified by PCR using primer sets with added restriction enzyme sequences. The DNA fragment obtained following digestion by the restriction enzymes was inserted into the retroviral expression plasmid pMYs-IRES-puro (Cell Biolabs, Inc., San Diego, CA). The correct sequence of the cDNA in the expression plasmid was confirmed by DNA sequencing.

### Cells and cell lines.

PXB cells were purchased from PhoenixBio (Hiroshima, Japan) and cultured according to the supplier’s protocol. Daudi lymphoma and HepG2 cell lines were obtained from the JCRB Cell Bank (JCRB9071) and ATCC (HB-8065), respectively. Huh7 cells were obtained from the JCRB Cell Bank (JCRB0403). Cell lines were cultured in Dulbecco’s modified Eagle’s medium (DMEM; (Thermo Fisher Scientific) or RPMI 1640 (Thermo Fisher Scientific) supplemented with 10% fetal bovine serum (FBS), 100 U/mL of penicillin, 100 μg/mL of streptomycin, and 100 U/mL of nonessential amino acids (Thermo Fisher Scientific).

### Transfection.

To generate human NTCP-expressing cells, Daudi, HepG2, or Huh7 cells were transduced with a retroviral vector encoding NTCP or Flag tag-NTCP with 10 μg/mL of Polybrene (Sigma-Aldrich, St. Louis, MO). The retroviral vectors were generated by modification of a previously described method (31). Briefly, the retroviral expression plasmid and the envelope expression plasmid p10A1 were cotransfected into GP2-293 cells using the Retro-X universal packaging system (TaKaRa Bio Inc., Shiga, Japan). At 2 days posttransfection, the culture supernatants were filtered through 0.45-μm filters and then concentrated using a Retro-X concentrator (TaKaRa Bio USA). At 48 h postinfection, cells were subjected to an immunofluorescence assay or a viral infection assay. HepG2-NTCP and HepG2/Flag-NTCP cells were maintained in DMEM/F-12, GlutaMAX supplement (catalog no. 10565018; Thermo Fisher Scientific) supplemented with 10% fetal calf serum (FCS) in the presence of 50 μM hydrocortisone, 5 μg/mL of insulin, and puromycin (0.5 μg/mL; InvivoGen).

### NTCP protein purification and reconstitution into liposomes.

Human NTCP proteins were expressed in High Five insect cells (Thermo Fisher Scientific) using the Bac-to-Bac baculovirus expression system (Thermo Fisher Scientific), and cultured cells were disrupted by homogenization. The membrane fraction was isolated and solubilized with 1% dodecylmaltoside (DDM; Anatrace, Maumee, OH). Solubilized proteins were purified with StrepTactin affinity beads (StrepTactin Sepharose; GE Healthcare, Chicago, IL), an anion-exchange column (HiTrap Q HP; GE Healthcare), and a size exclusion column (Superose 6 Increase; GE Healthcare) in Tris-HCl buffer (pH 7.0) containing 0.1 M NaCl, 0.05% DDM, and 0.002% cholesteryl hemisuccinate (Anatrace). Purified proteins were reconstituted into liposomes consisting of egg phosphatidylcholine with the adjuvant lipid A (50, 51).

### Immunization.

NTCP KO mice were subcutaneously primed with hNTCP (30 μg/mouse) in TiterMax Gold (TiterMax USA, Inc.). Two weeks later, the mice were alternately immunized in the abdominal cavity with NTCP liposomes (50 μg/mouse) or hNTCP transfectants (2 × 10^7^/mouse) at 2-week intervals. hNTCP transfectants were treated with gamma rays (10 Gy) prior to immunization. Splenic B cells were purified from immunized mice after a total of 4 to 6 immunizations and used for hybridoma preparations. As an adjuvant in immunization, poly(I·C) (Sigma) was used for immunization with hNTCP liposomes, and ODN 1826 (vaccine grade; InvivoGen, San Diego, CA) was used for immunization with NTCP transfectants. All experiments were performed following protocols approved by the RIKEN Animal Care and Use Committee.

### Enrichment of IgG^+^ B cells from immunized mice.

IgG^+^mature B cells were separated by negative selection using the MACS system (Miltenyi Biotec, Bergisch Gladbach, Germany). Unwanted mononuclear cells (T cells, NK cells, monocytes, dendritic cells, granulocytes, and IgM^±^ B cells prior to B cell receptor class switching) were targeted via a biotin-labeled antibody cocktail specific for the cell surface receptor(s) of those cell types. IgG^+^mature B cells were specifically enriched from the sample without retention in the LS column in the presence of a magnetic field (Miltenyi Biotec).

### Cell fusion and hybridoma selection.

IgG^+^ B cells enriched from the spleens of immunized mice were mixed with P3U1 myeloma cells at a 1:1 ratio and subjected to electrofusion using a Nepagene ECFG21 electroporator (Nepa Gene Co. Ltd., Ichikawa, Chiba, Japan) according to the manufacturer’s instructions. The cells were then seeded at 3 × 10^3^ to 1 × 10^4^/well in a 96-well flat-bottom plate and selected in HAT (sodium hypoxanthine, aminopterin and thymidine) medium. When cells covered half the bottom of a well, their culture supernatants were tested by flow cytometry for reactivity with NTCP^+^ Daudi transfectants but not the parental cell line. A portion of the hybridoma cells derived from positive culture supernatants was frozen in CELLBANKER 1 plus (TaKaRa bio). The remaining cells were subcultured until confluent, and then each supernatant was tested for inhibition of HBV infection.

### Flow cytometry screening.

For screening, a Daudi cell line expressing NTCP and one expressing green fluorescent protein (GFP) but not NTCP were mixed at a 1:1 ratio. Cells were then stained with the hybridoma supernatant and stored on ice for 20 min, followed by incubation with allophycocyanin (APC)-conjugated anti-mouse IgG (BioLegend). Cells were washed and resuspended in propidium iodide (Sigma-Aldrich) and analyzed for NTCP staining using an LSRFortessa X-20 (BD Biosciences, Franklin Lakes, NJ).

### Screening of hybridoma supernatants.

The hybridoma supernatants were screened by flow cytometry to detect NTCP-positive antibodies. A total of 792 positive hybridoma supernatants were identified as positive hits.

Anti-HBV assays using recombinant HBV encoding the NanoLuc gene (HBV/NL) were conducted as previously reported (33). Briefly, HepG2-NTCP-myc cells (5 × 10^4^/well) were pretreated with 100 μL of positive hybridoma supernatant in 96-well plates for 2 h and then infected with HBV/NL. At 24 h postinfection, cells were washed 3 times with phosphate-buffered saline (PBS). At 8 days postinfection, the level of NanoLuc activity and the host cell viability were determined using a Nano-Glo luciferase assay system (Promega, Madison, WI) and a CellTiter-Glo 2.0 cell viability assay system (Promega), respectively.

### Cloning of hybridomas.

Hybridomas of interest were recovered from the frozen cell stock, cultured in HT (sodium hypoxanthine and thymidine) medium overnight, and then cloned by limiting dilution (0.3 cell/well). Clones generally appeared in 8 to 15 days, and 20 to 30 clones from a single cloning were again screened for antibody reactivity and inhibition of HBV infection. Subcultures with the highest titers were then cloned by limiting dilution (0.3 cell/well) and screened again using the same procedure.

### Cell adaptation to serum-free medium and production of MAbs.

Acclimatization to serum-free medium was achieved through a two-step process. In the first step, hybridoma cell lines were adapted to culture in 10% FCS, HT-free, BM-Condimed H1-free RPMI 1640 medium. Thereafter, the cells were adapted to culture in serum-free PFHM-II medium (Thermo Fisher Scientific) and transferred to CELLine flask culture (BD Biosciences) according to the manufacturer’s instructions. After 14 days of culturing at 37°C and 5% CO_2_, the cells and the medium were collected from the cell culture chamber.

Monoclonal antibodies in the collected culture supernatants were purified by protein G Sepharose 4 Fast Flow (GE Healthcare) and stored at 4°C following sterile filtration.

### Synthesis of lipopeptides.

Alexa Fluor 647-coupled myristoylated HBV-preS1 lipopeptide and its mutant were synthesized by GlyTech, Inc. (Kyoto, Japan) with reference to the report by Meier et al. (52). In the mutant, the l-leucine at position 11 and the l-phenylalanine at position 13 were replaced by the respective d-enantiomers. The d-Leu^11^, d-Phe^13^ preS1 (isomer) was inactive in binding to NTCP (51).

### [^3^H]taurocholate uptake inhibition by N6HB426-20.

[^3^H]taurocholate uptake experiments were performed as previously reported (14). In brief, cells growing in 48-well plates were incubated for 30 min with N6HB426-20 or preS1 peptide at different doses and then washed twice with Na^+^ Ringer solution. Following preincubation for 10 min in Na^+^ Ringer solution, cells were incubated with 1 μM [^3^H]taurocholate for 15 min at 37°C, washed extensively with chilled PBS, and then lysed with 100 μL of 1% Triton X-100 in water for 10 min. The lysate was mixed with 900 μL of liquid scintillation cocktail (Ultima Gold XR; Perkin-Elmer, USA) followed by liquid scintillation counting (ALOKA liquid scintillation counter LSC-6100; Hitachi, Tokyo, Japan).

### HBV preparation.

HBV was derived from the culture supernatant of HepAD38 cells (kindly provided by Christoph Seeger at the Fox Chase Cancer Center). The cells were maintained in DMEM/F-12–GlutaMAX (Thermo Fisher Scientific) supplemented with 10% FBS, 100 U/mL of penicillin, 100 μg/mL of streptomycin, 5 μg/mL of insulin, and 500 ng/mL of tetracycline.

The collected supernatants were filtered through a 0.45-μm filter (Merck Millipore, MA) and concentrated approximately 500 times using polyethylene glycol 6000 (PEG 6000; Sigma-Aldrich).

### HBV entry inhibition assay.

HepG2 cells stably expressing human NTCP-myc in a 96-well plate were treated with preS1 peptide, anti-ovalbumin (anti-OVA) MAb, or N6HB426-20 MAb for 1 h and then washed with PBS. Cells were infected with HBV at a concentration of 500 genome equivalents per cell in the presence of 4% PEG 8000 and 2% dimethyl sulfoxide (DMSO) along with the preS1 peptide or each antibody. At 24 h postinfection, cells were washed with PBS and cultured in fresh medium containing 2% DMSO. At 12 days postinfection, the level of HBeAg was determined by enzyme-linked immunosorbent assay (ELISA; Abnova, Taipei, Taiwan).

Alternatively, PXB cells were treated with preS1 peptide, anti-OVA MAb, or N6HB426-20 MAb for 1 h and washed with PBS. Cells were infected with HBV (genotype C or genotype D derived from HBV-infected patients) at a concentration of 10 genome equivalents per cell in the presence of 4% PEG 8000 and 2% DMSO, along with preS1 peptide or each antibody. At 24 h postinfection, cells were washed with PBS. HBV-infected cells were cultured in fresh medium containing 2% DMSO. At day 7 postinfection, the level of HBsAg was determined by a chemiluminescent enzyme immunoassay (Lumipulse f, Fujirebio, Japan).

### Human hepatocyte chimeric mouse experiments.

Generation of uPA^+/+^ SCID^+/+^ mice and transplantation of human hepatocytes were performed as previously described (33). All mice were transplanted with frozen human hepatocytes obtained from the same donor. Ten chimeric mice, in which >90% of the liver tissue had been replaced by transplanted human hepatocytes, were inoculated via the tail vein with human serum containing 10^3^ copies of HBV. The chimeric mice were injected intravenously with 2.0 mg of either N6HB426-20 (*n* = 10) or normal mouse IgG (FUJIFILM Wako Pure Chemical Corporation, Osaka, Japan), as a control (*n* = 10), 1 day before and 2 days after HBV inoculation. In addition, they were injected intraperitoneally with 1.0 mg of each antibody at 1, 2, and 3 weeks after HBV inoculation. In the second experiment, the chimeric mice were injected intravenously with 1.0 mg of either N6HB426-20 MAb (*n* = 10) or anti-mouse OVA IgG MAb, as a control (*n* = 5), 1 day before and 1 day and 4 days after HBV inoculation. After inoculation, mouse sera were collected weekly. For quantitative analysis of HBV DNA, 10 μL of mouse serum was diluted with 690 μL of PBS and DNA was extracted. HBV DNA was quantified by real-time PCR using the cobas 6800 system (Roche Diagnostics KK, Tokyo, Japan). The lower quantitation limit of this assay is 3.61 log copies/mL. All animal protocols described in this study were performed in accordance with the *Guide for the Care and Use of Laboratory Animals* (53) and the local committee for animal experiments at Hiroshima University. Inoculation and extraction of serum samples were performed under isoflurane anesthesia. Human serum samples were obtained from a patient who provided written informed consent to participate in the study. Serum samples obtained from mice were aliquoted and stored in liquid nitrogen prior to use.

### Measurement of serum bile acid.

In the human hepatocyte chimeric mouse experiments, mouse sera were collected prior to MAb administration and weekly after HBV inoculation. Serum bile acid concentration was measured in an outsourcing laboratory (BML, Inc., Tokyo, Japan) by an enzyme cycling method using 10 μL of mouse serum after diluting it with 290 μL of PBS. Serum samples obtained from mice were aliquoted and stored in liquid nitrogen prior to use.

### N6HB426-20 epitope mapping.

Regions of extracellularly exposed N terminus and extracellular loops were determined as results of prediction of transmembrane helices in NTCP using TMHMM Server v. 2.0 (https://services.healthtech.dtu.dk/service.php?TMHMM-2.0; data not shown). Streptavidin-coated 96-well plates (Thermo Scientific; 436014) were used to capture each peptide at 10 μg/mL in PBS at room temperature for 2 h and washed five times with 0.1% Tween 20/PBS (PBST). Wells were then blocked with 200 μL of Nacalai Bullet Blocking One for 30 min. Recombinant N6HB426-20 mIgG2a MAb and control mouse IgG (Equitech-Bio; SLM66) were diluted into 5% Nacalai Bullet Blocking One in PBST. After discarding the blocking reagent solution, wells were incubated with 100 μL of antibody-diluted solution (1 μg/mL of IgG in PBST) overnight at 4°C and then washed five times with PBST. Bound IgG was detected with 100 μL of horseradish peroxidase-conjugated rabbit anti-mouse IgG (Dako; P0260) diluted 1:1,0000 in PBST for 3 h at room temperature and then washed five times with PBST. One-step Ultra TMB-ELISA (100 μL/well; Thermo Scientific; 34028) was added to the wells and allowed to develop at room temperature for several minutes. The reaction was stopped with 100 μL of 2 M H_2_SO_4_. Optical density (OD) was read at 450 nm on a microplate reader (Bio-Rad; model 680).
